# Sweet relief-endoscopic ultrasound-guided jejunojejunostomy using a lumen-apposing metal stent for candy cane syndrome

**DOI:** 10.1055/a-2971-3992

**Published:** 2026-10-19

**Authors:** Hinrich Fehrendt, Hauke Hamel, Franziska Halfmann, Nikolaus Schäfer, Maximilian Bockhorn, Christian Meinhardt, Mark Ellrichmann

**Affiliations:** 1 Department of Internal Medicine, GastroenterologyUniversity Medical Center OldenburgOldenburgGermany; 2 Department of General and Visceral SurgeryUniversity Medical Center OldenburgOldenburgGermany

Candy cane syndrome (CCS) is an uncommon but increasingly recognized complication after gastrectomy. 1​ An elongated blind jejunal limb preferentially fills with enteric contents, resulting in stasis, progressive dilatation, and compression of the efferent limb. Patients typically present with dysphagia, regurgitation, abdominal pain, and early satiety. 2​ Although surgical resection is the standard treatment, endoscopic ultrasound (EUS)–guided jejunojejunostomy using a lumen-apposing metal stent (LAMS) offers a minimally invasive alternative. 3​ ​4​

A 66-year-old patient underwent subtotal gastrectomy with Roux-en-Y reconstruction for gastric signet-ring cell carcinoma following neoadjuvant FLOT chemotherapy (initial tumor stage ypT3N0). After local tumor recurrence, extensive salvage surgery including completion gastrectomy and esophagojejunostomy was performed (rpT4bN1 G3).

Several weeks later, the patient developed severe dysphagia, odynophagia, and regurgitation of undigested food. Upper endoscopy revealed ulcerative reflux esophagitis and a markedly dilated blind jejunal limb causing compression of the efferent jejunal loop, consistent with CCS. Contrast-enhanced computed tomography confirmed the diagnosis and excluded recurrent malignancy ( Fig. 1 ).

**Fig. 1:**
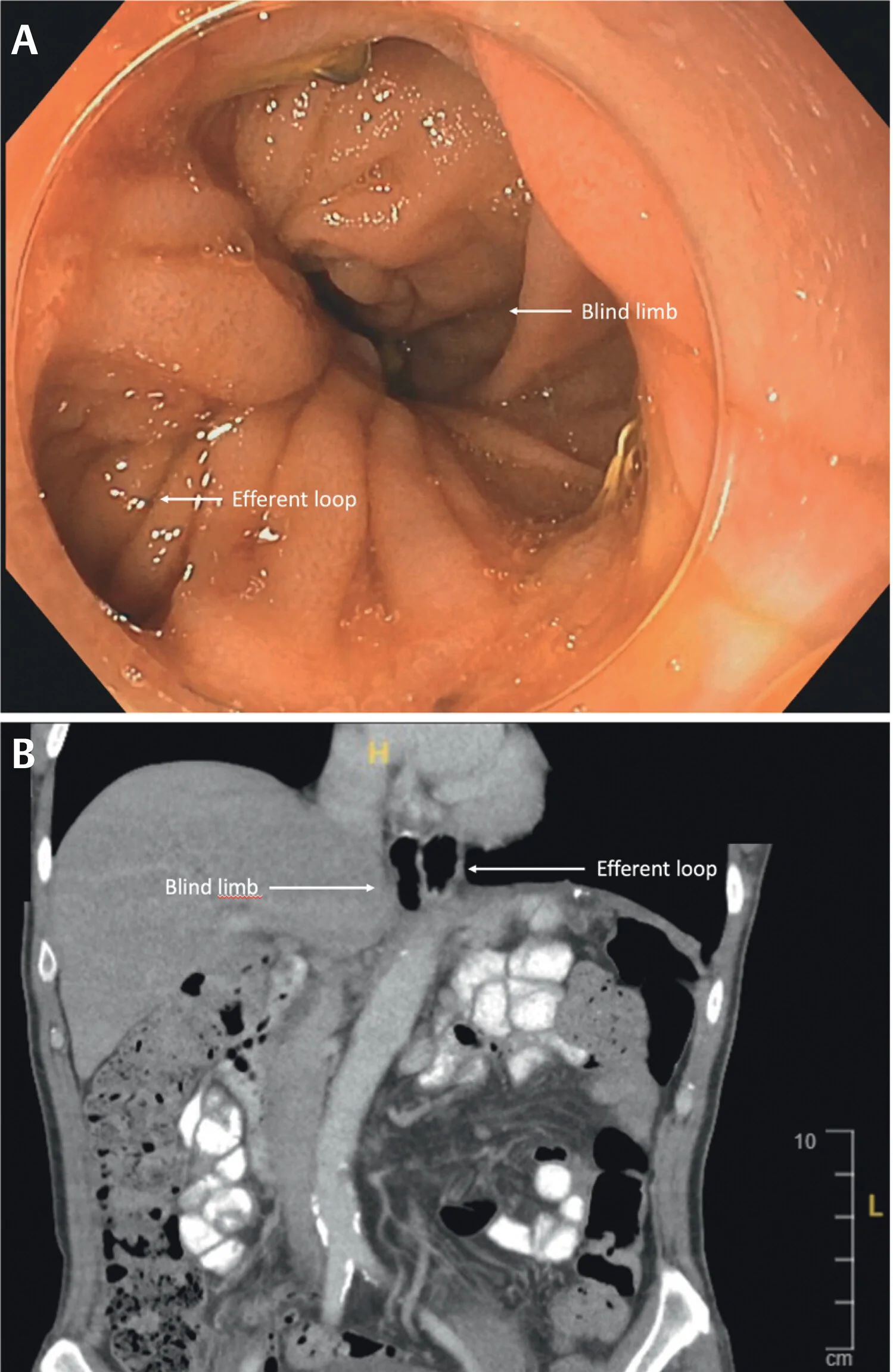
( **A** ) An endoscopic view to E/S-esophago-jejunostomy with a blind limb. ( **B** ) CT scan showing “candy cane syndrome.”

Given the patient's high operative risk, endoscopic treatment was performed. A guidewire was advanced into the efferent jejunal loop, followed by the placement of a gastric tube for water instillation. After intravenous butylscopolamine, a linear echoendoscope was introduced into the blind limb. The adjacent water-filled efferent loop was identified by EUS, and a direct jejunojejunostomy was created using an electrocautery-enhanced LAMS of 15×10 mm. Immediate decompression of the blind limb and restoration of intestinal flow were confirmed endoscopically ( Video 1 ). The patient's symptoms resolved rapidly, allowing the prompt resumption of oral nutrition ( Fig. 2 ).

**Video 1** Endoscopic ultrasound–guided Jejunojejunostomy using a lumen-apposing metal stent for candy cane syndrome. URI for the video:


**Fig. 2:**
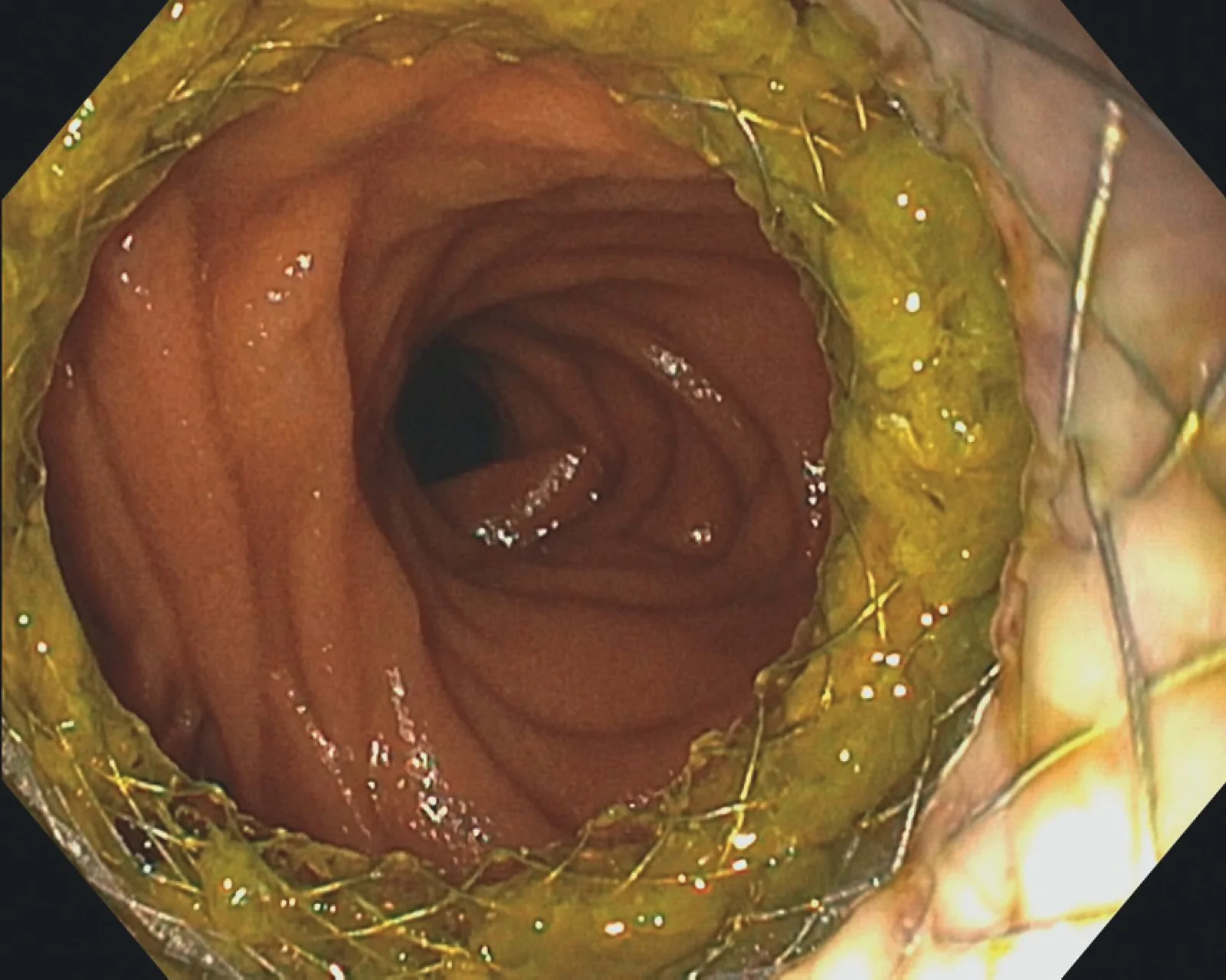
An endoscopic view through the LAMS into the adjacent efferent jejunal loop.

This case demonstrates that EUS-guided LAMS-assisted jejunojejunostomy is a feasible and effective minimally invasive treatment for CCS, providing rapid symptom relief while avoiding high-risk surgical revision in patients with complex postoperative anatomy.

Endoscopy_UCTN_Code_TTT_1AO_2AZ

## Declaration of GenAI Use

No generative artificial intelligence (AI) tools were used in the preparation of this manuscript or the associated figures, illustrations, clinical images, procedural video footage, or other submitted materials. Generative AI was also not used for writing, editing, or modifying the text. All contents were created, reviewed, and approved solely by the authors.
